# Acinetobacter baumannii Early-Onset Sepsis After Home Delivery Into Toilet Water

**DOI:** 10.7759/cureus.45951

**Published:** 2023-09-25

**Authors:** Patrícia Sousa, Beatriz Sousa, Francisca Calheiros-Trigo, Mariana Martins, Clara Paz-Dias

**Affiliations:** 1 Department of Pediatrics, Hospital Senhora da Oliveira, Guimarães, PRT; 2 Department of Neonatology, Hospital Senhora da Oliveira, Guimarães, PRT

**Keywords:** nosocomial epidemiology, hospital infection control, pediatrics & neonatology, acinetobacter baumanii, early onset neonatal sepsis

## Abstract

Early-onset sepsis (EOS) is an important cause of morbidity and mortality in newborns, usually caused by pathogens acquired intrapartum.

We present the case of a term neonate born by home delivery in the toilet, after an unsupervised pregnancy. He developed a culture-proven early-onset sepsis caused by *Acinetobacter baumannii*. This was the first case of neonatal sepsis by this pathogen in our unit. The microorganism was susceptible to all antibiotics tested. The neonate was treated empirically with ampicillin and cefotaxime and completed 21 days of directed therapy with meropenem, as meningitis could not be excluded. During the clinical course, the newborn developed severe and persistent thrombocytopenia and neutropenia.

In this report, we discuss the etiology behind this clinical presentation. We intend to raise awareness for the consideration of *Acinetobacter baumannii* as a potential pathogen in EOS, particularly in the presence of adverse birth circumstances.

## Introduction

Early-onset sepsis (EOS) affects approximately 0.5 to 18.5 per 1,000 live births and is most frequently caused by organisms acquired intrapartum, the most common of which is *Group B Streptococcus* (GBS) [1,2]. Preterm infants are at a higher risk due to immune system immaturity and diminished skin barrier function, but term neonates are also susceptible [3].

The improvement in perinatal care in recent decades has contributed to a decrease in incidence, but this is still an important cause of morbidity and mortality in the neonatal period [1,4]. Neurodevelopmental sequelae are a particular concern in these individuals [5]. In term neonates, the mortality rate is 2-3%, proving higher in preterm infants and in non-industrialized countries [5].

This case was previously presented in a "Meet the Expert" session at the 41st Annual Meeting of the European Society for Paediatric Infectious Diseases (ESPID) on May 12, 2023.

## Case presentation

A male neonate with an unsupervised pregnancy was born by eutocic home delivery in the toilet. According to the mother, the neonate was submerged for approximately two minutes while she called for help; the neonate was retrieved by the maternal grandmother. When paramedics arrived, the neonate was found connected to the umbilical cord and cyanotic. Upon hospital admission, he presented with polycythemia due to late cord clamping, hypothermia, and hypoglycemia. He was admitted to the neonatal intensive care unit (NICU) and stabilized. Gestational age was estimated at 38-39 weeks according to the Ballard score. The mother was 19 years old, obese, and reported tobacco and alcohol use during pregnancy. Maternal history was otherwise unremarkable, with no history of sexually transmitted diseases or HIV exposure. Forty-eight hours later, the neonate became pale and presented with prolonged capillary refill time and temperature instability. There were no signs of hemorrhage, skin lesions, or dysmorphic features. Laboratory results revealed mild thrombocytopenia (135.000/uL) and an elevated C-reactive protein (CRP) (110.7 mg/L) and excluded anemia and leukopenia. Coagulation tests were normal. Blood culture was obtained and lumbar puncture was performed but cerebrospinal fluid (CSF) was not obtained. Following the suspicion of EOS with a possibility of meningitis, empirical antibiotic therapy with ampicillin and cefotaxime was started. On the fifth day, there was no clinical improvement and *Acinetobacter baumannii complex* was isolated from blood culture. CSF was obtained but blood-contaminated and the antibiotic regimen was switched to meropenem. The pathogen identified in a total of three blood cultures was sensitive to all tested antibiotics (meropenem, imipenem, ciprofloxacin, levofloxacin, amikacin, gentamicin, tobramycin, and trimethoprim/sulfamethoxazole). CSF culture was negative. Despite the overall improvement, with clinical resolution, normal hematocrit, decreasing CRP and subsequently negative blood cultures, severe thrombocytopenia persisted (minimum 4000/μL), requiring seven platelet transfusions and one dose of intravenous immunoglobulin. There was no maternal thrombocytopenia and maternal platelet antibodies were negative. Because the father’s identity was not confirmed, further immunologic and genetic studies were unfeasible.

On the tenth day of life, temperature instability recurred and a maculopapular skin rash developed. Virologic real-time polymerase chain reaction panel (RT-PCR) on the nasal secretions (testing adenovirus, influenza A and B viruses, parainfluenza 1, 2, and 3 viruses, respiratory syncytial virus, human metapneumovirus, and coronavirus OC43) was negative, as were cytomegalovirus, Epstein Barr virus, herpes simplex virus 1/2, and human immunodeficiency virus serologies. The abdominal ultrasound was unremarkable. The epicutaneo-cava catheter was replaced and vancomycin was added. The following day, the patient developed neutropenia (minimum 500/μL).

On the fifteenth day, blood and catheter cultures were negative, thrombocytopenia had resolved and the patient had clinically improved, so vancomycin was suspended. Meropenem was continued for 21 days after the last positive blood culture. Neutropenia resolved by the twenty-seventh day of life. The patient was discharged home to be followed up by a neonatology consultant. At eight months of follow-up, there was no recurrence of thrombocytopenia or neutropenia and the infant presents with normal psychomotor development.

## Discussion

EOS is defined as a sepsis that occurs in the first 72 hours of life [5]. The most commonly implicated agents are acquired intrapartum when colonizers of the maternal genitourinary tract contaminate the amniotic fluid, placenta, cervix, or vaginal canal [3]. The most prevalent of which is GBS, particularly in term neonates [6]. Prenatal GBS colonization screening and antibiotic prophylaxis in selected cases have contributed to minimizing EOS incidence in recent years [1]. *Escherichia coli *is the second most frequent agent in term neonates and the first in preterms, usually presenting with a more severe clinical course and resulting in higher morbidity and mortality [7]. Additionally implicated pathogens are *Streptococcus pneumoniae*, *Staphylococcus aureus*, *Enterococcus spp.*, *Enterobacter spp.*, *Haemophilus influenzae*, and *Listeria monocytogenes* [8].

*Acinetobacter baumannii* is a rarely implicated agent in neonatal sepsis [9]. Most reported cases relate to late-onset sepsis, as this is almost exclusively a nosocomial agent [9]. Cases of EOS caused by *Acinetobacter baumannii* have been reported in India and South Africa but are not expected in industrialized countries [10,11].

This was the first case of either early or late-onset sepsis caused by *Acinetobacter baumannii* identified in Hospital Senhora da Oliveira NICU in Guimarães, Portugal. The unsanitary birth circumstances are likely the origin in this case [12]. Poor prenatal care and maternal substance abuse are also important risk factors associated with neonatal sepsis [3].

Thrombocytopenia etiology could not be completely clarified and was most likely multifactorial. Sepsis was a predominant factor, but the potentially adverse intrauterine environment or polycythemia could be the cause [13,14]. Auto- or alloimmune thrombocytopenia, caused by the transplacental passage of maternally derived antiplatelet antibodies, is less likely but could not be excluded [15,16].

Congenital neutropenia may be caused by decreased neutrophil production (e.g. maternal hypertension, bone marrow failure syndromes, inborn errors of metabolism) or increased neutrophil destruction (as occurs in immune-mediated neutropenia, in the presence of infection or when drug-dependent antibodies form against neutrophil membrane glycoproteins) [17]. In this case, neutropenia proved transient and may have been drug-induced, particularly in relation to treatment with meropenem. Nonetheless, meropenem was continued because this causative effect could not be confirmed and the benefits outweighed the risks.

## Conclusions

Early-onset sepsis caused by *Acinetobacter baumannii* is rare, mostly found in non-industrialized countries. This is the first case in our NICU. Despite being more frequently associated with nosocomial infection, the birth circumstances may have been the origin in this case and empirical treatment in similar situations should cover this microorganism, adjusted to local resistance patterns. Severe and prolonged thrombocytopenia could be sepsis-related or immune in etiology. Neutropenia was most likely caused by treatment with meropenem.
